# Efficacy and mechanism of steep pulse irreversible electroporation technology on xenograft model of nude mice: a preclinical study

**DOI:** 10.1186/s12957-018-1386-6

**Published:** 2018-04-25

**Authors:** Zuoqing Song, Xiaohong Xu, Minghui Liu, Jinghao Liu, Jun Chen, Chengxiang Li, Chenguo Yao, Qinghua Zhou

**Affiliations:** 1 Department of Lung Cancer Surgery, Tianjin Lung Cancer Institute, Tianjin Medical University General Hospital, Tianjin, 300052 China; 2 College of Nursing, Tianjin Medical University, Tianjin, 300070 China; 3 College of Electrical Engineering, Chongqing University, Chongqing, 400044 China

**Keywords:** Steep pulse, Large cell lung cancer, In vivo imaging system, Necrosis, Apoptosis, Caspase-3

## Abstract

**Background:**

Steep pulse therapy can irreversible electrically brackdown of tumor membrance and cause cell death. In previous studies, we investigated the effect of steep pulsed electroporation on the killing of large cell lung cancer cell line L981- in vitro, and determined the best parameters for killing lung cancer cells by steep pulse technology. But the optimal parameters and the mechanisms of steep pulse irreversible electroporation technology on nude mouse tumor model are unclear.

**Methods:**

Three settings of steep pulse therapy parameters were applied to the nude mouse model. An in vivo imaging system was employed to observe the effect of different parameters on the mouse model. The pathological changes of the tumor tissue and immunofluorescence data on Caspase-3 protein expression were recorded.

**Results:**

Under the in vivo imaging system, the steep pulse had an obvious inhibitory effect on the transplanted tumor in the nude mouse model. Pathological tests showed that occurrence of necrosis and apoptosis and expression of Caspase-3 protein in the tumor tissue were increased compared to those in the normal tissue.

**Conclusions:**

Steep pulse irreversible electroporation technology showed a promising antitumor effect in the nude mouse tumor model. With splint-type electrode, the best treatment parameters determined for the nude mouse tumor model were voltage amplitude 2000 V/cm, pulse width 100 μs, pulse frequency 1 Hz, pulse number 60, and repeat time 3. Moreover, steep pulse induced coagulative necrosis of tumor tissue by cell apoptosis.

## Background

In a previous study [1, 2], our research group had explored the efficacy of steep pulse electroporation technique to kill large cell lung cancer cell line L9981; we defined the optimal parameters for killing the lung cancer cells and discussed the mechanism of steep pulse technology in cancer cell death [3]. However, the application and outcome of the technology for tumor tissues and tumor cell lines may be quite different. Therefore, it is absolutely necessary to explore the effective killer pulse parameters in an animal model for tumor [4]. On the basis of previous studies [1, 2], we adopted large cell lung cancer cell line L9981 in a nude mouse tumor model as the research subject. We used an in vivo imaging system to explore the parameters in the nude mouse tumor model by steep pulse irreversible electroporation technology and studied the underlying mechanism of the process at the cellular level. This study can potentially provide a theoretical basis for future clinical trials.

## Methods

### Materials

Human highly metastatic large cell lung cancer cell lines L9981, which screened to establish through application of a single cell clones from human large cell lung cancer cell lines WCQH-9801, the large cell lung cancer cell lines L9981 have highly invasive and highly metastatic. And we uesd 30 female BALB/C nude mice which were 4–6 weeks old and weighted 17.5–20 g. Fluorescein was purchased from Shanghai Ruixing Gene Technology Co Ltd.; RPMI-1640 medium and newborn calf serum were purchased from GIBCO Co; Caspase-3 fluorescent immunoassay kit was purchased from Beijing Biyuntian Biological Reagent Company. Other conventional reagents were supplied by local companies. The study has been evaluated and approved by the veterinary ethical committee.

### Instruments and equipment

We used an energy-controllable steep pulse therapeutic apparatus, designed and manufactured by Chongqing University, China, which could combine the different pulse parameters and produce energy-controllable steep pulse by capacitor-mediated energy storage and discharge. The Xenogen in vivo imaging system (IVIS 200) was purchased from Xenogen, USA. Irreversible steep pulse apparatus and plate/pin-type electrodes were presented by State Key Laboratory of Power Transmission Equipment and System Security and New Technology, Chongqing University.

### Methods

#### Cell culture

Human large cell lung cancer cell line L9981-luc was cultured in RPMI-1640 medium including 10% fetal bovine serum at 37 °C and 5% CO_2_. When cell growth reached the confluence of approximately 80%, 0.125% trypsinized cells with 0.2% EDTA were routinely passaged.

#### Cell suspension in steep pulse technology

When cells were in a logarithmic growth phase and in good condition, 0.125% protease was added for digestion and percussed for a single cell suspension. The concentration of living cells was adjusted to 1 × 10^7^ cells/ml.

#### Establishment of tumor model in nude mice

**Fig. 1:**
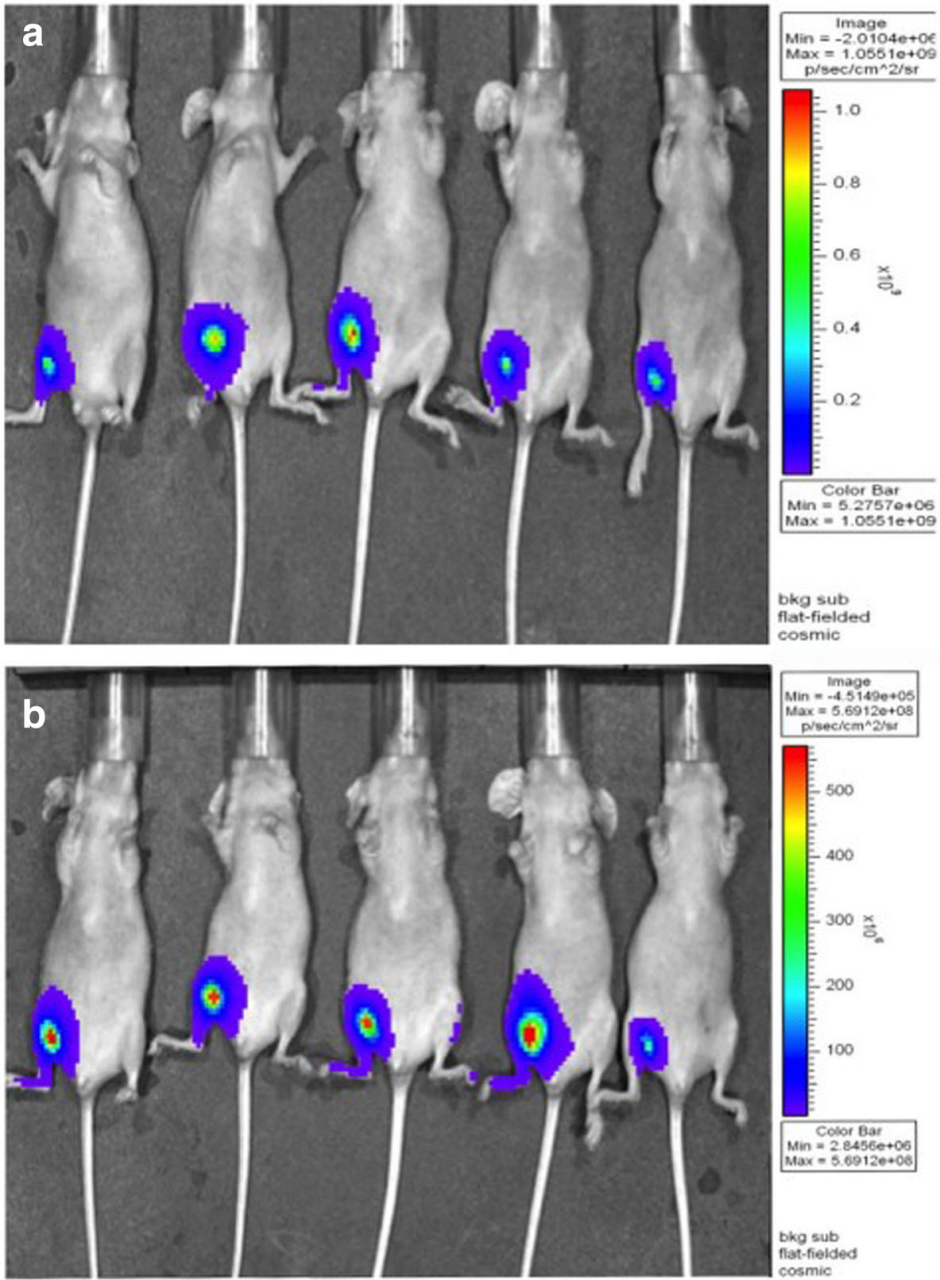
Pictures of materials. **a** The instant images of the nude mouse by vivo imaging system after subcutaneous injection of large cell lung cancer cell lines L9981-luc. **b** The observation of nude mouse subcutaneous transplanted tumor by vivo imaging system observation after 1 week

Nude mice were randomly grouped and housed in cages. Two hundred microliters of L9981-luc cell suspension (1 × 10^7^ cells/ml) was injected subcutaneously in the right hind groin of the nude mice after disinfection. The substrate (about 150 mg/kg, 0.2 ml) was injected into their abdominal cavity, and they were put into the anesthetic box of IVIS 200 system, using inhalation anesthesia by 3% isoflurane for 10 min. The subcutaneous transplantation tumor in the nude mouse was observed by an in vivo imaging system (Fig. 1a).

#### Feeding and observing the nude mouse models for tumor

Nude mice were bred conventionally and observed weekly using the in vivo imaging system after the subcutaneous injection of large cell lung cancer cell line L9981-luc. The mice conditions of the injection point, spirit, diet and defecation, body weight, and tumor volume were routinely observed. The time of tumor formation and tumor size were also recorded, and the next steps were followed once the maximum diameter of the tumor grew to approximately 0.8–1 cm (Fig. 1b).

#### Steep pulse parameter setting

The steep pulse parameter 1 setting was voltage amplitude 2000 V/cm, pulse width 100 s, pulse frequency 1 Hz, pulse number 30, and repeat time 1; parameter 2 was voltage amplitude 2000 V/cm, pulse width 100 s, pulse frequency 1 Hz, pulse number 60, and repeat time 2; and parameter 3 was voltage amplitude 2000 V/cm, pulse width 100 s, pulse frequency 1 Hz, pulse number 60, and repeat time 3. Parameter settings and operation steps of the control group were exactly the same as that of the experimental group, except for the use of steep pulse therapy machine in the latter.

#### Operation of steep pulse treatment in nude mouse tumor model

Approximately 150–200 μl of 0.8% pentobarbital was administered according to the mouse weight (40 mg/kg). After intraperitoneal injection of the anesthetic, the mouse was put in the IVIS 200 system for pre-treatment observation of tumor signal. The nude mouse was fixed after 30 min; steep pulses were given in accordance with parameter 1, parameter 2, and parameter 3, respectively. The same operation was applied to the control group, without turning the steep pulse therapy machine on. After steep pulse treatment, intraperitoneal injection of fluorescein was given. Ten minutes later, the nude mouse was put in the IVIS 200 system to observe the post-treatment signal. Thereafter, the nude mouse was returned to its cage and continued with conventional breeding. Dynamic observation of in vivo imaging was performed at 24 and 72 h. The nude mouse was executed thereafter, and the tumor bodies were removed and fixed with 10% formalin for histopathological observation.

#### Effect of steep pulse therapy on large cell lung cancer cell line L9981 in nude mouse model for tumor

Tumor growth time and tumor size were continuously observed and recorded. For the experimental group, the major axis and minor axis of the tumor were measured periodically, and the tumor volume was calculated. The weight of the nude mouse was measured periodically and recorded.

#### Qualitative analysis of Caspase-3 protein expression by immunofluorescent staining

Tissue slices were taken from both experimental group and control group for qualitative detection. Conventional de-waxing and hydration, antigen retrieval with microwave, and immunofluorescent staining were performed, followed by qualitative analysis under the microscope.

## Results

### Differences between pre-treatment and post-treatment signal intensities in parameter 1 setting

**Fig. 2:**
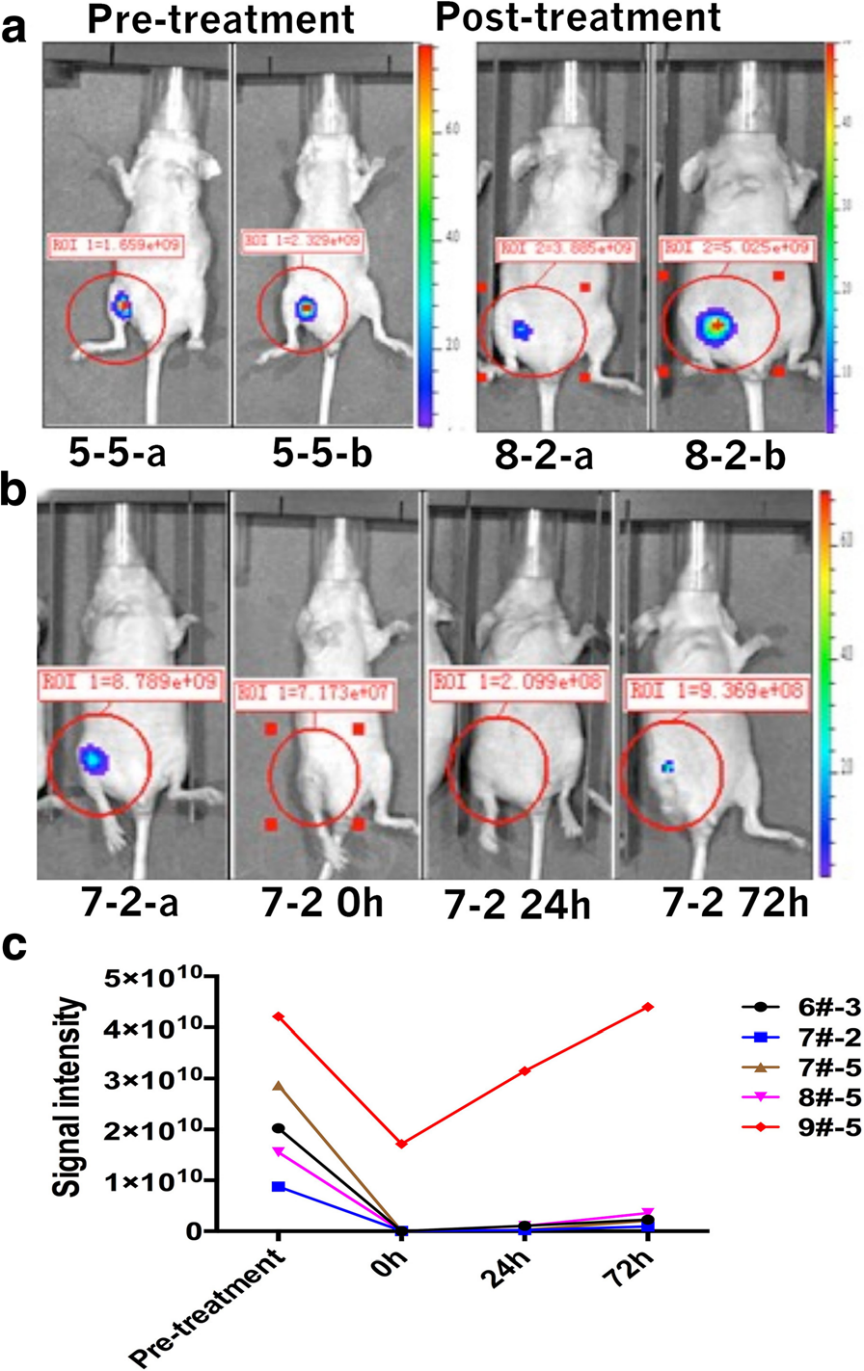
Differences between pre-treatment signal intensity and post-treatment signal intensity of parameter 1. a Differences between pre-treatment signal intensity and post-treatment signal intensity in the negative control group of nude mouse tumor. b Differences between pre-treatment signal intensity and post-treatment signal intensity of parameter 1 in nude mouse tumor. c The trend of pre-treatment signal intensity and post-treatment signal intensity of parameter 1 in nude mouse tumor

The mice were randomly grouped, and the differences between pre-treatment and post-treatment signal intensities were recorded in the IVIS 200 system (Tables 1 and 2; Fig. 2a–c).

**Table 1:** Differences between pre-treatment signal intensity and post-treatment signal intensity of parameter 1 in the negative control group of nude mouse tumor

Nude mouse numbered	3–4	5–1	5–5	8–2
Pre-treatment signal intensity	9.662e+09	2.394e+08	1.659e+09	3.885e+09
After 72-h signal intensity	2.301e+10	2.595e+08	2.329e+09	5.025e+09

The signal intensity values in the table is the size of ROI (region of interest) included the total flux, unit: p/s

**Table 2:** Differences between pre-treatment signal intensity and post-treatment signal intensity of parameter 1 in the IRE group of nude mouse tumor

Nude mouse numbered	Pre-treatment signal intensity	Instant-treatment signal intensity	After 24-h signal intensity	After 72-h signal intensity
6#-3	2.025e+10	3.635e+06	1.060e+09	2.291e+09
7#-2	8.789e+09	7.173e+07	2.099e+08	9.369e+08
7#-5	2.870e+10	4.016e+07	2.669e+08	2.072e+09
8#-5	1.550e+10	3.340e+05	1.031e+09	3.566e+09
9#-5	4.211e+10	1.713e+10	3.150e+10	4.397e+10

The signal intensity values in the table is the size of ROI (region of interest) included the total flux, unit: p/s

LSD test showed statistically significant differences between pre-treatment and post-treatment signal intensities (*P* = 0.024). The differences between pre-treatment and 24-h post-treatment signal intensities were also statistically significant (*P* = 0.016). However, those between 24 h post-treatment and 72 h post-treatment signal intensities were not statistically significant. Similarly, the differences between immediate post-treatment and 72 h post-treatment signal intensities were also not statistically significant.

### Differences between pre-treatment and post-treatment signal intensities in parameter 2 setting

**Fig. 3:**
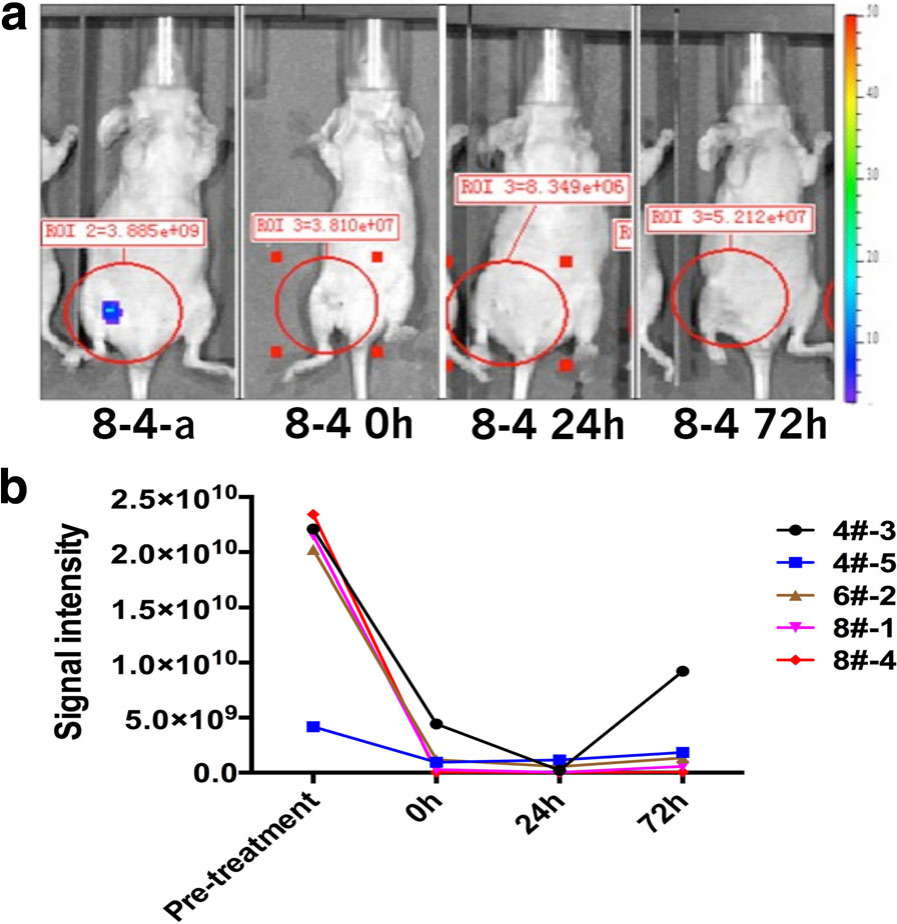
Differences between pre-treatment signal intensity and post-treatment signal intensity of parameter 2. **a** Differences between pre-treatment signal intensity and post-treatment signal intensity of parameter 2 in the IRE group of nude mouse tumor. **b** The trend of pre-treatment signal intensity and post-treatment signal intensity of parameter 2 in nude mouse tumor

The mice were randomly grouped, and differences between pre-treatment and post-treatment signal intensities were observed in the IVIS 200 system (Table 3; Fig. 3a–b).

**Table 3:** Differences between pre-treatment signal intensity and post-treatment signal intensity of parameter 2 in the IRE group of nude mouse tumor

Nude mouse numbered	Pre-treatment signal intensity	Instant-treatment signal intensity	After 24-h signal intensity	After 72-h signal intensity
4#-3	2.210e+10	4.423e+09	2.169e+08	9.211e+09
4#-5	4.182e+09	9.476e+08	1.166e+09	1.852e+09
6#-2	2.025e+10	1.184e+09	5.598e+08	1.351e+09
8#-1	2.150e+10	2.839e+08	3.018e+07	5.714e+08
8#-4	2.343e+10	3.810e+07	8.349e+06	5.212e+07

The signal intensity values in the table is the size of ROI (region of interest) included the total flux, unit: p/s

Least significant difference (LSD) test showed statistically significant differences between the pre-treatment and post-treatment signal intensities (*P* = 0.000). The differences between pre-treatment and 24-h post-treatment signal intensities (*P* = 0.000) and those between pre-treatment and 72-h post-treatment signal intensities were also statistically significant.

### Differences between pre-treatment and post-treatment signal intensities in parameter 3 setting

**Fig. 4:**
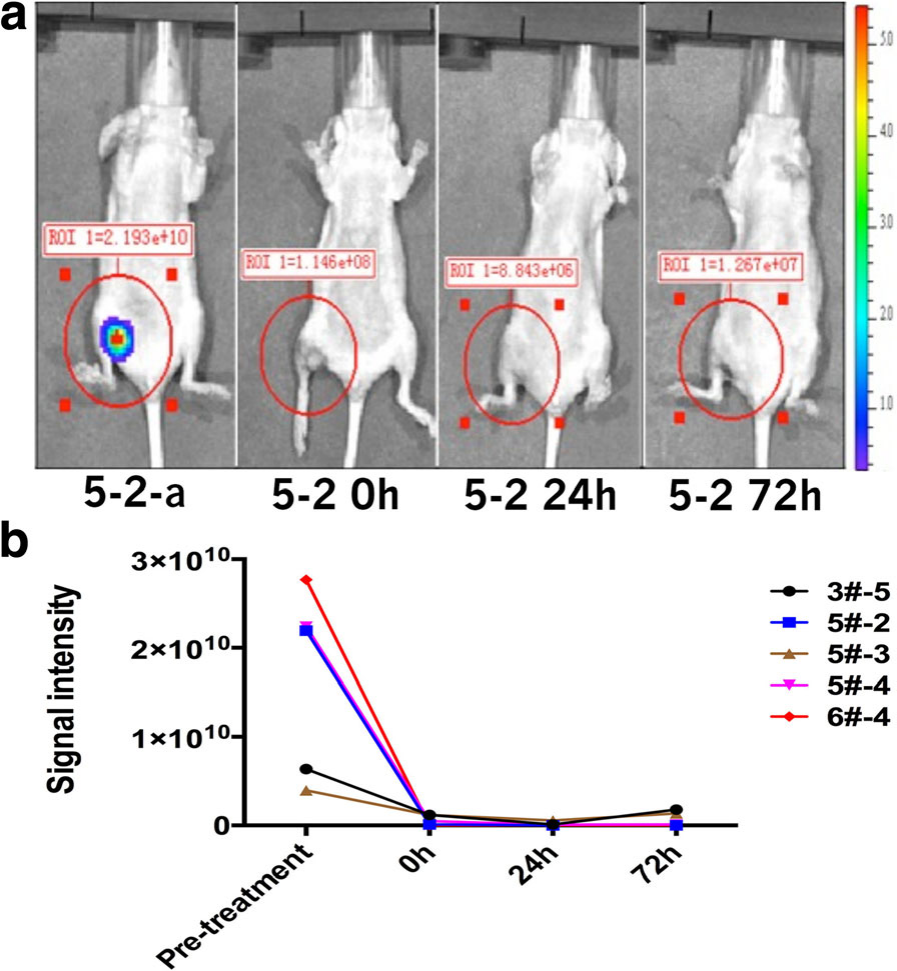
Differences between pre-treatment signal intensity and post-treatment signal intensity of parameter 3. **a** Differences of pre-treatment signal intensity and post-treatment signal intensity of parameter 3 in the IRE group of nude mouse tumor. **b** The trend of pre-treatment signal intensity and post-treatment signal intensity of parameter 3 in nude mouse tumor

As earlier, the differences between pre-treatment and post-treatment signal intensities were observed in IVIS 200 system (Table 4; Fig. 4a–b).

**Table 4:** Differences between pre-treatment signal intensity and post-treatment signal intensity of parameter 3 in the IRE group of nude mouse tumor

Nude mouse numbered	Pre-treatment signal intensity	Instant-treatment signal intensity	After 24-h signal intensity	After 72-h signal intensity
3#-5	6.359e+09	1.188e+09	1.246e+08	1.781e+09
5#-2	2.193e+10	1.146e+08	8.843e+06	1.267e+07
5#-3	3.951e+09	3.624+07	7.765+06	1.243+06
5#-4	2.238e+10	4.787e+08	8.527e+07	1.091e+08
6#-4	2.768e+10	1.405e+07	2.733e+06	8.122e+06

The signal intensity values in the table is the size of ROI (region of interest) included the total flux, unit: p/s

Rank sum test results showed *χ*^2^ = 12.234, *P* = 0.007, and statistical significance of the differences between pre-treatment and post-treatment signal intensities was of the order: pre-treatment > immediately post-treatment > 24-h post-treatment > 72-h post-treatment.

### Observed effects of steep pulse therapy on large cell lung cancer cell line L9981 in nude mice

The tumor growth time and size, along with the mouse weight, were recorded periodically, and the tumor volume was calculated. The corresponding data have been shown in Tables 5 and 6.

**Table 5:** The volume changes of transplantation tumors after treatment of steep pulse ($\overline{x}\pm s$, *n* = 9)

Group	1st day (mm^3^)	5th day (mm^3^)	12th day (mm^3^)
Treatment group	47.88 ± 27.53	18.08 ± 13.68	10.82 ± 11.06
Control group	62.66 ± 7.78	60.00 ± 28.34	64.82 ± 50.09

**Table 6:** The comparison of volume, weight, and inhibition rate in nude mouse subcutaneous transplantation tumor by steep pulse technology

Group	Tumor weight (g)	Weight inhibition rate	Tumor volume (cm^3^)	Volume inhibition rate
Treatment group	0.644 ± 0.271	68.6%	0.441 ± 0.359	73.5%
Control group	2.048 ± 0.836		1.667 ± 0.702	

Tumor volume—*V* = *ab*2/2, *a* = the longest tumor diameter, *b* = short tumor diameter

Volume(weight) inhibition rate:{*V*(weight)control − *V*treatment(weight)} ÷ *V*(weight)control × 100%

With SPSS 16.0 analysis software, independent sample rank sum test was applied to the experimental group: *χ*^2^ = 7.220, *P* = 0.02; the comparison of the first day with the 12th day was *Z* = − 2.402, *P* = 0.016, which implied statistical significance. Analysis of variance of factorial design showed (1) comprehensive analysis: *F* = 3.789, *P* = 0.013; (2) inter-group analysis: *F* = 13.248, *P* = 0.002; and (3) time analysis: *F* = 1.232, *P* = 0.312. Regarding the comparison of weight, *P* = 0.009 implied statistical significance, and regarding the comparison of volume, *P* = 0.011 showed statistical significance. Comparison of distant metastasis between experimental and control groups showed no statistical significance.

### Gross observation of steep pulse therapy on large cell lung cancer cell line L9981 in nude tumor mouse

During the steep pulse therapy, respiration, heart range, and other vital functions of the nude mouse were stable, and all mice could tolerate the entire procedure quite well. With irreversible electroporation (IRE) treatment, mild twitches were seen in the nude mice. After the experiment, the mice showed decreased appetite. Most of them were drowsy with less movement. Few of them were found irritable. However, all the symptoms recovered to pre-treatment conditions in 2–3 days.

### Visual observation related to steep pulse therapy on large cell lung cancer cell line L9981 in nude tumor mouse

Nude mouse tumor was located in the subcutaneous tissues, with clear border separating the surrounding tissues. The average maximum diameter was 8–10 mm. The tumor section appeared like a grayish-red solid and was capsuled. During IRE treatment, a black necrotic tissue was noticed in the anode region. The anode region was better than the cathode in hemostasis. Bubbles were noticed in the cathode region with less necrosis. Tumor sections prepared immediately after the procedure were soft, grayish white in appearance, and marked with blood drops. Over time, scars formed, volume decreased, and congestive necrosis became visible. Later on, eschar fell off the necrotic tumor, the skin was completely recovered, and no palpable tumor remained under the skin.

### Light microscopy observation of steep pulse therapy for large cell lung cancer cell line L9981 in nude tumor mouse

**Fig. 5:**
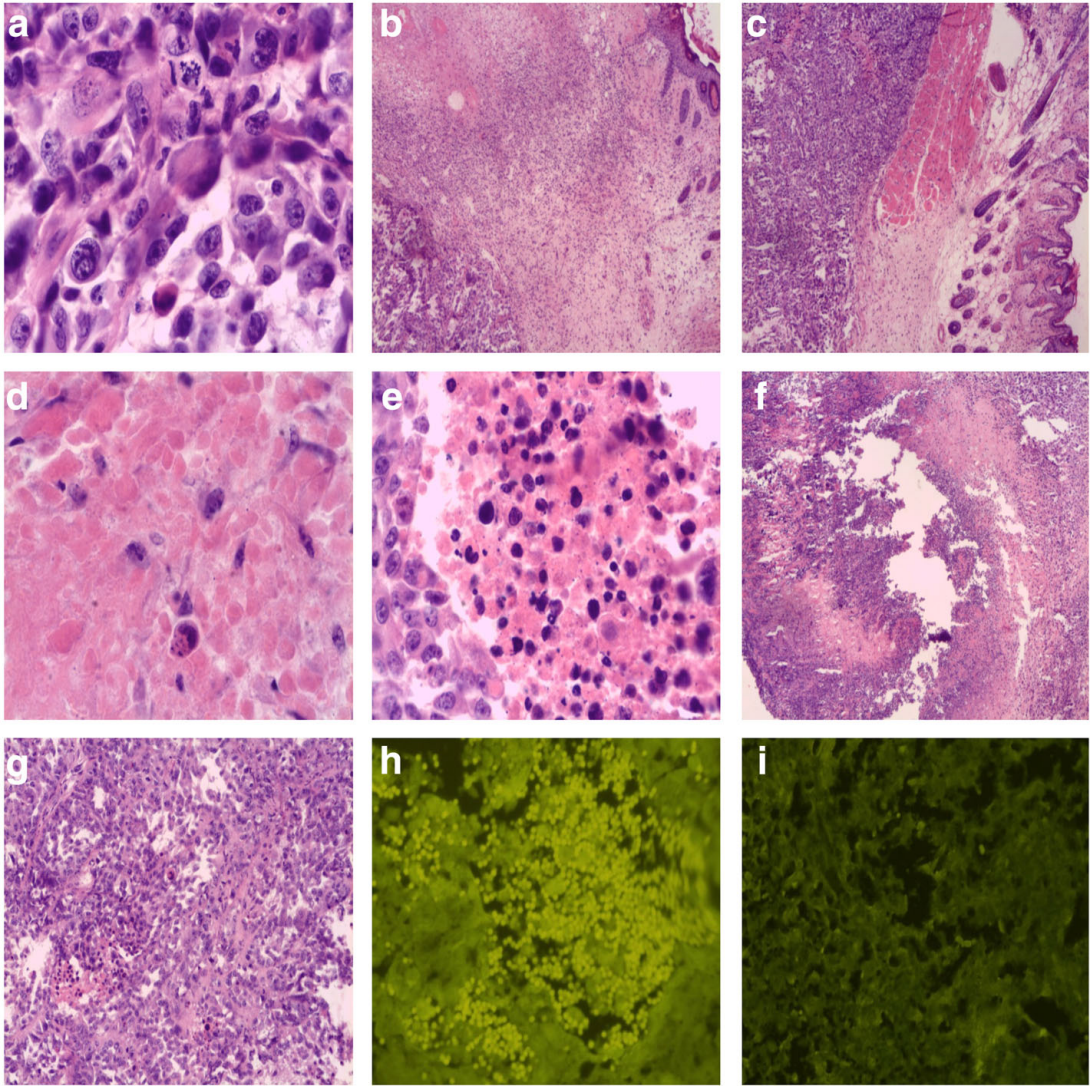
Light microscope observation of steep pulse therapy for large cell lung cancer cell line L9981 in nude tumor mouse and qualitative analysis of caspase-3 protein expression with immunofluorescent staining. **a** Multinuclear, meganucleus, prominent nucleoli, and relatively more mitoses were noticed in control group (× 400). **b** A homogeneous red-stained patch was noticed in the anode region, with blurred cell structure, and appeared with coagulation necrosis (× 400). **c** Cell structures of cathode were still clear with no significant changes, so as the deep subcutaneous tumor cells (× 400). **d** Tumor cell necrosis, darkening of cytoplasm eosin staining, dark staining of karyopyknosis, cytoplasm eosin staining, and nuclear pyknosis hyperchromatic (× 400). **e** Necrosis border was clear, with karyopyknosis, karyorrhexis, and karyolysis (× 400). **f** Tumor tissue necrosis, obviously, there are large necrosis area (× 400). **g** Karyopyknosis, karyorrhexis, and karyolysis (× 400). **h** Yellow fluorescence spotty distribution of caspase-3 by fluorescence microscopy. **i** Experimental group caspase-3 expression by fluorescence microscopy was obviously more than the control group

The control group had an actively growing tumor, with high density and less intercellular substance. The tumor cells appeared with a sheet- or nest-like distribution with obvious atypia. Multinuclear, meganuclear, or cells with prominent nucleoli, and with relatively more mitoses were noticed (Fig. 5a). The experimental group was observed to have significant differences in the histologic changes in the cathode and anode regions, close to the epidermis. A homogeneous red-stained patch was noticed in the anode region, with blurred cell structure and coagulative necrosis (Fig. 5b); cell structures in the cathode region were clearer with no significant change, like the deep subcutaneous tumor cells (Fig. 5c). Seventy-two hours after treatment, different levels of necrosis appeared in the deep subcutaneous tumor cells, with darkening of eosin-stained cytoplasm, darkened staining due to karyopyknosis, and a small patch of necrosis (Fig. 5d). Five days after IRE treatment, tumor tissues were drawn, and HE staining was performed. Necrosis was found in the electrode areas, around which different levels of inflammatory cell infiltration were noticed. The necrosis border was clear, with karyopyknosis, karyorrhexis, and karyolysis (Fig. 5e); twelve days after the treatment, necrosis was visible in the whole tumor, and a large area of necrosis was noticed, with karyopyknosis, karyorrhexis, and karyolysis (Fig. 5f, g).

### Qualitative analysis of Caspase-3 protein expression with immunofluorescent staining after steep pulse therapy for large cell lung cancer cell line L9981 in nude tumor mouse

After immunofluorescent staining, spotty distribution of yellow fluorescence was noticed under the fluorescence microscope, and the staining in the experimental group was obviously more than that in the control group (Fig. 5h, i).

## Discussion

Lung cancer is a kind of malignant tumor, which has the highest incidence and mortality worldwide, and the incidence is still on the rise [3]. Non-small cell lung cancer (NSCLC) accounts for the majority of lung cancers, at approximately 80–85%, including squamous carcinoma, adenocarcinoma, and large cell carcinoma. Non-small cell lung cancer (NSCLC) is also a primary threat to the survival of patients with lung cancer. According to the data published by the World Health Organization (WHO) in 2005, the mortality rate in patients with lung cancer is 3.083/million (4.1343/million for males and 1.984/million for females), which tops the list for all malignant tumors. According to clinical data of 1742 Chinese patients with stage IV NSCLC, 1-year, 2-year, 3-year, 4-year, and 5-year survival rates were 44, 22, 13, 9, and 6%, respectively [4]. Towing to concealment of lung cancer, a large number of patients reach the last stage before they reach the hospital and miss the stage of surgical recovery. In recent years, there has been a significant improvement in diagnostic technology and comprehensive therapeutic approaches including surgery, radiotherapy, and chemotherapy. However, no obvious improvement could be achieved in the long-term survival rate of patients with lung cancer. Therefore, devising comprehensive therapeutic approaches for lung cancer treatment is imperative.

In recent years, minimally invasive ablation, aiming to inactivate tumor cells and elimination of tumor load, has drawn much attention. In addition, the widely applied innovative targeted-ablation therapies have achieved great progress in the treatment of patients who missed the surgical option. The innovative targeted-ablation therapies include cryosurgery [5, 6], high-intensity focused ultrasound [7, 8], radioactive particle implantation [9, 10], microwave [11, 12] or radiofrequency ablation [13], and other physically targeted therapies. In malignant tumor treatment, compared to conventional therapies such as chemotherapy, radiotherapy, and physiotherapy, the remarkable advantage was seen in the use of steep pulse, owing to its irreversible electric puncture [14]. Steep pulse therapy has the potential of becoming a newly localized physiotherapy for tumor and effectively complementing the comprehensive treatment options for tumor.

Steep pulse technology is a combination of biology, new electrotechnics, microelectronics, and many other disciplines. As a new potential approach for tumor treatment, steep pulse technology has captured academic attention in·the world. The development of steep pulse is rapidly emerging [15]. Its main mechanism involves irreversible electric puncture with instantaneous high-voltage electric pulse stimulation to produce temporary and reversible microholes on the cell membrane. When the electric pulse is stopped, most of the microholes are closed, affecting the cells [16]. With increasing dosage of an electric pulse, both the membrane and nucleus are in a dynamic process of charging and discharging, an electric field is formed surrounding the membrane, eventually leading to cell death by irreversible electric puncture [17–19]. In recent years, studies on the steep pulse technique showed its encouraging prospects in tumor treatment.

Our preliminary study optimized the parameters for steep pulse therapy in nude tumor mouse: voltage amplitude 2000 V/cm, pulse width 100 s, pulse frequency 1 Hz, pulse number 10, and repeat times 6. With the above parameters, a maximum number of tumor cells could be killed. Presently, in biotechnological applications, increasing number of studies are focusing on “steep pulse electric field-induced apoptosis of tumor cells.”

Apoptosis refers to gene-controlled, programmed, and active cell death, which is also called programmed cell death (PCD). Studies show that apoptosis is closely related to tumorigenesis and tumor regression [20, 21]. Induction of a large number of tumor cells to undergo apoptosis and initiation of the programmed active cell death procedure would be a major breakthrough in antitumor treatment. Therefore, seeking such a novel treatment is the highlight of tumor treatment research today.

Currently, in the medically advanced countries, such as those in Europe and the USA, real-time monitoring of the pathological processes of tumorigenesis and tumor development, by using in vivo bioluminescence imaging, is an important and rapidly developing frontier area of research.

Compared to conventional tumor tests, the use of in vivo bioluminescence imaging to observe steep pulse irreversible electric puncture in nude mice offers the following advantages: (1) high-sensitivity—IVIS imaging system (purchased from Xenogen company) can detect tumors comprising only a few hundred cells. A diameter of 1 mm is the minimal dimension, composed of around 10^6^–10^7^ cells, that can be detected by the currently used methods. Therefore, the IVIS imaging system is several thousand times more sensitive than the conventional methods. (2) High-precision—fluorescence value can be obtained immediately after inoculation; the results show the absolute value of the number of photons emitted by target cell per unit time, which has a direct linear correlation with the target cell number. The fluorescence value is objective, easy to analyze, and can precisely analyze the therapeutic effect quantitatively. (3) Self-control—the same results may be observed at different times, which can help understand the functional process of IRE treatment better in the nude tumor mouse.

In this experiment, we tracked the procedure of steep pulse irreversible puncture for large cell lung cancer cell line L9981 in nude tumor mouse. We noticed the changes after IRE treatment and determined the optimal parameters that can achieve the satisfactory antitumor effect—voltage amplitude 2000 V/cm, the pulse width 100 s, pulse frequency 1 Hz, pulse number 10, and repeat time 3.

After IRE treatment, we observed coagulative necrosis, karyopyknosis, karyorrhexis, karyolysis, and other necrotic signs in the tumor. Therefore, IRE could eventually lead to tumor apoptosis by activating the Caspase-3 protease in tumor cells.

This study showed the antitumor effect of IRE treatment; however, there is no marked difference in terms of tumor metastasis and recurrence, which implies that the IRE treatment available currently still cannot be used to prevent tumor metastasis and recurrence. Compared to high-intensity focused ultrasound, radiofrequency ablation, cryosurgery, and other local therapies, IRE possesses the following advantages: shorter duration, avoids heating effect, and includes the whole treatment area with an electric field. The above advantages provide great potential in comprehensive tumor treatment. As a new therapeutic approach, further studies would be required to identify the issues pertaining to biomedical security, electric parameters, and long-term effects. Unremitting efforts are required to promote IRE for clinical treatment.

According to our current experimental results, we hold the opinion that presently, satisfactory antitumor effects in large cell lung cancer cell line L9981 in nude tumor mice could be achieved using the following steep pulse treatment: voltage amplitude 2000 V/cm, pulse width 100 μs, frequency 1 Hz, pulse 60, and repeat times 3. After immunofluorescence staining of the post-treatment tumor section, spotty distribution of yellow fluorescence was noticed under the fluorescence microscope, which was obviously more abundant than in the control group. Steep pulse is able to activate the Caspase-3 protease in tumor cells, thereby inducing apoptosis of tumor cells, and eventually leading to tumor cell death. Due to the lack of accurate theoretical guidance regarding the actual distribution of electric field produced by the electrodes, the 3-D positioning of the electrode needle could not be made accurately. In addition to affecting the experimental results, it could also not ensure the complete coverage of the tumor with the electric field. However, the residual tumor might continue growing, probably faster, eventually inducing tumor recurrence. Although IRE technology can cause coagulative necrosis of tumor cells and effectively kill the tumor, it cannot control tumor metastasis and recurrence, or completely control the tumor.

## Conclusions

Steep pulse irreversible electroporation technology has potent antitumor effects on nude mouse tumor model. With a splint-type electrode, the best treatment parameters for the nude mouse tumor model were determined: voltage amplitude 2000 V/cm, pulse width 100 μs, pulse frequency 1 Hz, pulse number 60, and repeat time 3. Steep pulse induced coagulative necrosis of tumor tissue by cell apoptosis. Therefore, through this study, IRE technology may be a new approach to the treatment of cancer in the future.
